# Association of Cost Sharing With Delayed and Complicated Presentation of Acute Appendicitis or Diverticulitis

**DOI:** 10.1001/jamahealthforum.2021.2324

**Published:** 2021-09-03

**Authors:** Andrew P. Loehrer, Mary M. Leech, Julie E. Weiss, Chad Markey, Erik Wengle, Joshua Aarons, Stephen Zuckerman

**Affiliations:** 1 Department of Surgery, Dartmouth-Hitchcock Medical Center, Lebanon, New Hampshire; 2 Geisel School of Medicine at Dartmouth, Hanover, New Hampshire; 3 The Dartmouth Institute for Health Policy and Clinical Practice, Lebanon, New Hampshire; 4 Department of Biomedical Data Science, Geisel School of Medicine at Dartmouth, Lebanon, New Hampshire; 5 Urban Institute, Washington, DC

## Abstract

**Importance:**

Treatment delays are associated with increased morbidity and cost of disease, although the extent to which cost sharing influences timely presentation and management of acute surgical disease remains unknown. Given recent policy changes using cost sharing to modify health care behavior, this study examines the association of cost sharing with the health of the patient at presentation and with receipt of optimal or minimally invasive surgery.

**Objective:**

To assess whether cost sharing is associated with the likelihood of early, uncomplicated patient presentation or with surgical management of 2 representative emergency general surgery diagnoses: acute appendicitis and acute diverticulitis.

**Design, Setting, and Participants:**

This cohort study used Health Care Cost Institute claims from January 1, 2013, through December 31, 2017, to analyze data of commercially insured individuals hospitalized for acute appendicitis or diverticulitis. In total, 151 852 patients in the data set aged 18 to 64 years and presenting with acute appendicitis or diverticulitis were included as identified using the *International Classification of Diseases, Ninth Revision* and the *International Statistical Classification of Diseases and Related Health Problems, Tenth Revision*. Data were analyzed from January 2020 through February 2021.

**Exposures:**

The primary exposure was patient total cost sharing incurred for the index hospitalization, defined as their summed deductible, copayments, and coinsurance.

**Main Outcomes and Measures:**

The primary outcome was early, uncomplicated disease presentation. Secondary outcomes were receipt of optimal surgical care and minimally invasive surgery if undergoing an operation. Analyses were conducted with multivariable logistic regression models to adjust for patient characteristics and community-level socioeconomic and geographic factors. High cost sharing was defined as quartile 4 (>$3082), and low cost sharing as quartile 1 ($0-$502).

**Results:**

Among 151 852 patients, 52.4% were men, and the total cost-sharing median was $1725 (interquartile range, $503-$3082). Higher cost sharing was associated with lower odds of early, uncomplicated disease presentation (odds ratio, 0.63; 95% CI, 0.61-0.65). Patients with higher cost sharing were less likely to receive optimal surgical care (odds ratio, 0.96; 95% CI, 0.93-0.99) or minimally invasive surgery (odds ratio, 0.89; 95% CI, 0.84-0.95).

**Conclusions and Relevance:**

The findings of this cohort study suggest that, as policymakers debate the degree of cost sharing in public and private insurance plans, attention should be given to the clinical and financial implications associated with care delays.

## Key Points

**Question:**

Does an association exist between high cost-sharing insurance plans and patient presentation with and surgical management of acute appendicitis or acute diverticulitis?

**Findings:**

In this cohort study of 151 852 patients, higher patient cost sharing was associated with lower odds of presenting with early, uncomplicated disease, receiving optimal surgical care, and receiving minimally invasive surgery.

**Meaning:**

Policymakers should be aware of the clinical and financial implications of patient health care behaviors associated with increased cost sharing.

## Teaser

This cohort study uses commercial claims data to assess whether increased patient cost sharing is associated with the likelihood of early, uncomplicated presentation or surgical management of emergency general surgery diagnoses.

## Introduction

Access to timely care remains associated with socioeconomic disparities in surgical care. Previous work shows that delays in presentation with common acute, surgical conditions are associated with more complex presentation and less optimal surgical care.^1,2^ Insurance status has shown associations of time of disease presentation with odds of treatment delays owing to reduced financial barriers to care, among other factors.^3,4^ In particular, studies have shown that uninsured individuals have a higher probability of presenting with more complicated and severe emergency surgery diagnoses.^5^ Disease complexity and severity at the time of diagnosis are proportional to longer hospital stays, greater morbidity, and decreased probability of receiving a minimally invasive procedure.^5,6,7,8,9^ Conversely, increasing access to care through expanded insurance coverage has been associated with earlier presentation and improved management of surgical conditions.^10,11,12,13,14^ Both appendicitis and diverticulitis are common conditions cared for by emergency general surgery providers, including both the operative and nonoperative care of patients depending on their characteristics, such as age and comorbidities, as well as pathology-related factors, including severity of disease at the time of presentation.

However, far less is known about the association of patient cost sharing with presentation and management of common, costly, and morbid emergency general surgery conditions, such as acute appendicitis or diverticulitis. Private insurance plans are turning to cost sharing to increase patient responsibility, specifically by minimizing use of unwarranted care.^15,16^ In the seminal RAND Health Insurance Experiment,^17^ the introduction of cost sharing was a “blunt tool” that reduced the probability of patients seeking appropriate and inappropriate medical care without improving the overall quality of care. Conversely, the Oregon Experiment^18^ showed the expansion of low-cost preventive care use and reduction of high-cost emergency care with Medicaid expansion. Although prior studies have evaluated cost sharing in medical care, few studies have assessed the association between cost sharing and acute surgical conditions.^16^ Significant knowledge gaps remain regarding the degree to which patient cost sharing is associated with patient presentation and subsequent management of emergency general surgery diagnoses, especially acute appendicitis or diverticulitis.

The present study evaluated how cost sharing is associated with clinical presentation and care. The primary objective was to evaluate the degree to which patient cost sharing is associated with patient presentation with early, uncomplicated appendicitis or diverticulitis. The secondary objective was to determine whether the degree of cost sharing is associated with receipt of optimal surgical care or minimally invasive surgery. We hypothesized that high cost sharing is associated with decreased odds of presenting with early, uncomplicated disease.

## Methods

### Data and Cohort Development

**Figure 1.**
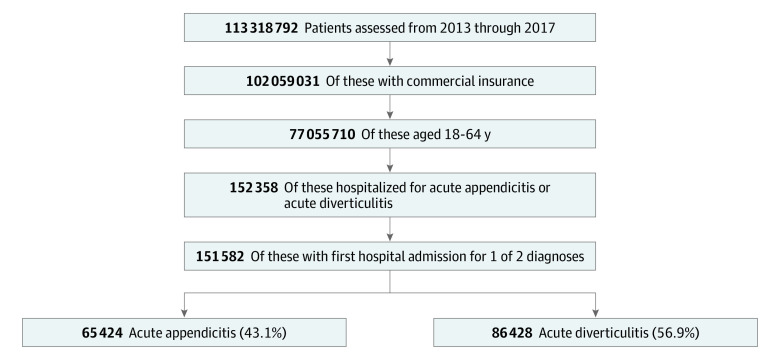
Patient Cohort Derivation

This cohort study used longitudinal commercial claims data from the Health Care Cost Institute (HCCI) to identify all patients with an index admission with acute appendicitis or acute diverticulitis from January 1, 2013, through December 31, 2017 (Figure 1). Since 2012, the HCCI data set included 1 billion claims per year, representing a population of 55 million per year from 4 health insurance companies (Aetna, Humana, Kaiser Permanente, and Blue Health Intelligence).^19^ Index hospitalizations were identified using principal *International Classification of Diseases, Ninth Revision* (*ICD-9*) and *International Statistical Classification of Diseases and Related Health Problems, Tenth Revision* (*ICD-10*) diagnosis codes (eTable 1 in the Supplement). Patients 18 to 64 years of age who were covered by private insurance through employee-sponsored plans, nongroup marketplace plans, and nonmarketplace individual plans were included. Patients were excluded if they were covered by Medicare or Medicare Advantage Plans given our focus on patients younger than 65 years and unique comorbidities of such patients with Medicare coverage. The index hospitalization was identified as the first admission with appendicitis or diverticulitis captured in claims data. The reporting of this study followed the Strengthening the Reporting of Observational Studies in Epidemiology (STROBE) reporting guideline for cohort studies. This study was deemed exempt from review by the Dartmouth-Hitchcock Medical Center.

### Outcomes

The primary outcome was presentation with early, uncomplicated disease at the time of admission. Consistent with prior work, early, uncomplicated presentation was defined as admission with nonruptured appendicitis or with diverticulitis (eTable 2 in the Supplement).^1,5,20,21^ Secondary outcomes included optimal surgical management and receipt of minimally invasive surgery. Optimal surgical management was defined as receipt of an appendectomy during admission for acute appendicitis or avoidance of colostomy or ileostomy when admitted with acute diverticulitis. Minimally invasive surgery was defined as receipt of a laparoscopic (vs open) procedure for patients undergoing either an appendectomy or sigmoid resection. Outcomes were defined from *ICD-9* and *ICD-10* diagnosis and procedure codes.

### Exposure

The primary exposure was the degree of cost sharing incurred for the index hospitalization, defined as the sum of the components of the patient’s coinsurance, copayment, and deductible. Total member cost sharing was defined as quartiles and expressed in US dollars. The low cost-sharing group was defined as being in quartile 1 ($0-$502), and the high cost-sharing group, in quartile 4 (>$3082). Sensitivity analyses were performed by setting a threshold of higher than $800 as being high cost sharing.

### Statistical Analysis

Frequency distributions (numbers and percentages) described the associations of patient characteristics, total cost sharing, type of diagnosis, socioeconomic deprivation, and geographic factors with outcomes. Distributions were calculated between characteristics or factors and total cost-sharing quartiles. Estimated odds ratios (ORs) and 95% CIs resulting from multivariable logistic regression models were used to assess the association of cost sharing with patient presentation and with surgical management. In addition, the trend in total cost sharing was estimated by treating total cost-sharing quartiles as being continuous. Models were adjusted for patient characteristics, including age, sex, calendar year, quarter at the time of hospital admission, regional location within 1 of 9 US Census Bureau geographic divisions, and a geographic rurality indicator.^22^ Furthermore, the models controlled for Social Deprivation Index, type of insurance benefit plan, and an indicator for enrollment in a high-deductible plan. Beneficiary 5-digit zip codes were used to establish rural-urban commuting area codes, consistent with methods used by the US Department of Agriculture.^23^ Rural was set as any area with rural-urban commuting area codes 4.0, 5.0, 6.0, 7.0, 7.2, 8.0, 8.2, 9.0, 2.1, 3.0, 4.1, 5.1, 7.1, 8.1, or 10.1. Using the patient’s state of residence, geographic region was calculated from the Census Bureau Region and Division Codes.^24^ Evaluation of local area measures of socioeconomic constraint used the Social Deprivation Index.^25^ The Social Deprivation Index was determined at the level of the zip code tabulation area and represented a composite of 9 variables, including employment, income, transportation access, and educational level, available through US Census Bureau data. The resultant index ranged from 0 to 100, with greater values representing a greater degree of community-level socioeconomic deprivation.

Sensitivity analyses included cost-sharing thresholds above and below $800, age group (25-64 years) subset, type of diagnosis, and a subset cohort (October 2015 through December 2017) to include adjustment for patient comorbidities. The Elixhauser comorbidity index was calculated from concurrent comorbid diagnoses (*ICD-10* codes) but were available only for patient claims from October 1, 2015, through December 2017.^26^

All analyses were performed from January 2020 through February 2021 using SAS, version 9.4 (SAS Institute Inc). A 2-sided value of *P* < .05 was considered statistically significant.

## Results

**Figure 2.**
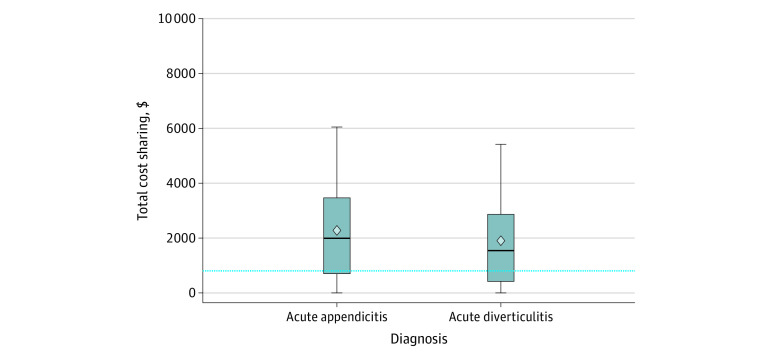
Boxplot of Total Cost Sharing by Diagnosis The horizontal line inside the box represents the median; the height of the box, the interquartile range; and the whiskers, the fifth and 95th interquartile ranges. The dashed horizontal line represents the threshold of $800.

Among 151 852 patients (52.4% men and 47.6% women), the total cost-sharing median was $1725 (interquartile range [IQR], $503-$3082). By diagnosis, the median cost sharing per episode of appendicitis (43.1%) was $1992 (IQR, $700-$3446) and for acute diverticulitis (56.9%), $1524 (IQR, $421-$2865) (Figure 2). The majority of patients resided in urban areas (91.5%) and had low-deductible plans (74.9%). The most popular benefit plan type was point of service (69.0%), followed by health maintenance organization (12.6%), preferred provider organization (11.7%), exclusive provider organization (5.9%), and other, including indemnity and short-term plans (0.8%) (Table 1). Compared with patients in the low total cost-sharing group ($0-$502), patients in the high cost-sharing group (>$3082) were younger (<55 years), were without comorbidities, participated in a high-deductible plan, were hospitalized in the first quarter of the calendar year, carried a preferred provider organization benefit plan, or lived in the West South Central or Mountain region (eTable 3 in the Supplement). In the highest total cost-sharing quartile (>$3082), a steady increase was observed in the percentage of patients per year from 2013 through 2017 (20.9% to 29.0%).

**Table 1.** Total Cost Sharing, Diagnosis, and Patient Characteristics by Early, Uncomplicated Presentation; Optimal Surgical Care; and Minimally Invasive Surgery Among 151 852 Patients, 2013-2017

Characteristic^a^	Total, No. (%)	No. (%)					
		Early, uncomplicated presentation (n = 151 852)		Optimal surgical care (n = 151 852)		Minimally invasive surgery (n = 67 954 [44.7%])^b^	
		No	Yes	No	Yes	No	Yes
Total patients	151 852	43 377 (28.6)	108 475 (71.4)	58 054 (38.2)	93 798 (61.8)	12 419 (18.3)	55 535 (81.7)
Total cost sharing, $							
0-502	37 957 (25.0)	9204 (24.3)	28 753 (75.7)	14 994 (39.5)	22 963 (60.5)	3029 (19.9)	12 200 (80.1)
503-1725	37 981 (25.0)	9565 (25.2)	28 416 (74.8)	15 904 (41.9)	22 077 (58.1)	2766 (17.6)	12 958 (82.4)
1726-3082	37 954 (25.0)	11 323 (29.8)	26 631 (70.2)	14 212 (37.4)	23 742 (62.6)	3157 (17.8)	14 535 (82.2)
>3082	37 960 (25.0)	13 285 (35.0)	25 675 (65.0)	12 944 (37.1)	25 016 (65.9)	3467 (18.0)	15 842 (82.0)
Diagnosis							
Acute appendicitis	65 424 (43.1)	21 494 (32.9)	43 930 (67.1)	9462 (14.5)	55 992 (85.5)	7440 (13.3)	48 522 (86.7)
Acute diverticulitis	86 428 (56.9)	21 883 (25.3)	64 545 (74.7)	48 592 (56.2)	37 836 (43.8)	4979 (41.5)	7013 (58.5)
Age group, y							
18-24	11 281 (7.4)	2660 (23.6)	8621 (76.4)	1753 (15.5)	9528 (84.5)	1022 (10.9)	8374 (89.1)
25-34	18 459 (2.2)	4798 (26.0)	13 661 (74.0)	4709 (25.5)	13 750 (74.5)	1550 (12.1)	11 251 (87.9)
35-44	27 637 (18.2)	8205 (29.7)	19 432 (70.3)	10 818 (39.1)	16 819 (60.9)	2268 (16.7)	11 306 (83.3)
45-54	43 770 (28.8)	13 224 (30.2)	30 546 (69.8)	19 331 (44.2)	24 439 (55.8)	3559 (21.7)	12 875 (78.3)
55-64	50 705 (33.4)	14 490 (28.6)	36 215 (71.4)	21 443 (42.3)	29 262 (57.7)	4020 (25.5)	11 729 (74.5)
Sex							
Male	79 517 (52.4)	25 124 (31.6)	54 393 (68.4)	30 240 (38.0)	49 277 (62.0)	6712 (18.9)	28 843 (81.1)
Female	72 335 (47.6)	18 253 (25.2)	54 082 (74.8)	27 814 (38.5)	44 521 (61.5)	5707 (17.6)	26 692 (82.4)
Calendar year							
2013	35 407 (23.3)	8231 (23.3)	27 176 (76.7)	12 524 (35.4)	22 883 (64.6)	3277 (18.3)	14 663 (81.7)
2014	30 414 (20.0)	7863 (25.9)	22 551 (74.1)	11 776 (38.7)	18 638 (61.3)	2513 (17.9)	11 549 (82.1)
2015	28 542 (18.8)	8055 (28.2)	20 487 (71.8)	11 169 (39.1)	17 373 (60.9)	2247 (17.6)	10 526 (82.4)
2016	30 204 (19.9)	9837 (32.6)	20 367 (67.4)	11 782 (39.0)	18 422 (61.0)	2324 (18.9)	9986 (81.1)
2017	27 285 (18.0)	9391 (34.4)	17 894 (65.6)	10 803 (39.6)	16 482 (60.4)	2058 (18.9)	8811 (81.1)
Calendar quarter							
January through March	36 585 (24.1)	9956 (27.2)	26 629 (72.8)	13 454 (36.8)	23 131 (63.2)	3134 (18.5)	13 773 (81.5)
April through June	39 471 (26.0)	11 129 (28.2)	28 342 (71.8)	15 153 (38.4)	24 318 (61.6)	3256 (18.4)	14 439 (81.6)
July through September	39 056 (25.7)	11 306 (29.0)	27 750 (71.0)	15 408 (39.5)	23 648 (60.5)	3002 (17.3)	14 361 (82.7)
October through December	36 740 (24.2)	10 986 (29.9)	25 754 (70.1)	14 039 (38.2)	22 701 (61.8)	3027 (18.9)	12 962 (81.1)
Elixhauser comorbidity index^c^							
0	22 549 (41.3)	8014 (35.5)	14 535 (64.5)	7408 (32.8)	15 141 (63.2)	2120 (16.7)	10 565 (83.3)
1	15 311 (28.1)	5399 (35.3)	9912 (64.7)	6369 (41.6)	8942 (58.4)	1194 (20.7)	4576 (79.3)
2	9230 (16.9)	2945 (31.9)	6285 (68.1)	4053 (43.9)	5177 (56.1)	609 (24.7)	1860 (75.3)
3+	7498 (13.7)	1941 (25.9)	5557 (74.1)	3040 (40.5)	4458 (59.5)	370 (28.5)	927 (71.5)
Social Deprivation Index							
Q1 (1-25)	49 536 (33.7)	14 657 (26.6)	34 879 (70.4)	19 149 (38.7)	30 387 (61.3)	3942 (17.7)	18 297 (82.3)
Q2 (26-50)	38 850 (26.5)	11 137 (28.7)	27 713 (71.3)	14 850 (38.2)	24 000 (61.8)	3124 (18.0)	14 223 (82.0)
Q3 (51-75)	32 226 (22.0)	9047 (28.1)	23 179 (71.9)	12 256 (38.0)	19 970 (62.0)	2758 (19.2)	11 610 (80.8)
Q4 (76-100)	26 181 (17.8)	7164 (27.4)	19 017 (72.6)	9596 (36.6)	16 585 (63.4)	2151 (18.2)	9690 (81.8)
Rural							
Urban	134 341 (91.5)	38 429 (28.6)	95 912 (71.4)	50 673 (37.7)	83 688 (62.3)	10 546 (17.4)	50 031 (82.6)
Rural	12 458 (8.5)	3574 (28.7)	8884 (71.3)	5181 (34.2)	7277 (58.4)	1429 (27.4)	3791 (72.6)
Census Bureau geographic division							
New England	5489 (3.7)	1727 (31.5)	3762 (68.5)	2391 (43.6)	3098 (56.4)	427 (19.2)	1799 (80.8)
Middle Atlantic	21 886 (14.9)	5701 (26.1)	16 185 (73.9)	7640 (34.9)	14 246 (65.1)	1787 (17.1)	8649 (82.9)
East North Central	20 556 (14.0)	6317 (30.7)	14 239 (69.3)	8675 (42.2)	11 881 (57.8)	1894 (23.1)	6299 (76.9)
West North Central	9264 (6.3)	2983 (32.2)	6281 (67.8)	3720 (40.2)	5544 (59.8)	855 (21.1)	3207 (78.9)
South Atlantic	34 614 (23.6)	9446 (27.3)	25 168 (72.7)	13 922 (40.2)	20 692 (59.8)	2659 (18.9)	11 399 (81.1)
East South Central	7069 (4.8)	1935 (27.4)	5134 (72.6)	2959 (41.9)	4110 (58.1)	716 (26.2)	2017 (73.8)
West South Central	26 302 (17.9)	7287 (27.7)	19 015 (72.3)	9437 (35.9)	16 865 (64.1)	1973 (15.7)	10 626 (84.3)
Mountain	9546 (6.5)	3234 (33.9)	6312 (66.1)	3602 (37.7)	5944 (62.3)	690 (15.3)	3812 (84.7)
Pacific	12 065 (8.2)	3374 (28.0)	8691 (72.0)	3503 (29.0)	8562 (71.0)	974 (13.9)	6012 (86.1)
High-deductible plan							
No	113 186 (74.9)	31 568 (27.9)	81 618 (72.1)	43 476 (38.4)	69 710 (61.6)	9203 (18.4)	40 959 (81.6)
Yes	37 959 (25.1)	11 593 (30.5)	26 366 (69.5)	14 310 (37.7)	23 649 (62.3)	3129 (18.0)	14 293 (82.0)
Benefit plan type							
Point of service	104 737 (69.0)	30 447 (29.1)	74 290 (70.9)	38 946 (37.2)	65 791 (32.8)	8634 (18.0)	39 421 (82.0)
PPO	17 804 (11.7)	4852 (27.3)	12 952 (72.7)	7472 (42.0)	10 332 (58.0)	1504 (19.8)	6091 (80.2)
HMO	19 200 (12.6)	5249 (27.3)	13 951 (72.7)	7552 (39.3)	11 648 (60.7)	1568 (19.3)	6549 (80.7)
EPO	8926 (5.9)	2530 (28.3)	6396 (71.7)	3286 (36.8)	5640 (63.2)	653 (16.6)	3293 (83.4)
Other^d^	1185 (0.8)	299 (25.2)	886 (74.8)	798 (67.3)	387 (32.7)	60 (24.9)	181 (75.1)

Abbreviations: EPO, exclusive provider organization; HMO, health maintenance organization; PPO, preferred provider organization; Q, quartile.

^a^ Numbers missing were Social Deprivation Index, 5059; rurality, 5053; Census Bureau geographic division, 5061; and high deductible plan, 707.

^b^ Among patients with an appendectomy or colectomy.

^c^ Study cohort for this index included 54 588 patients (36.0%), and study period was October 2015 through 2017.

^d^ Other includes indemnity, short-term, and other.

Overall, rates of early, uncomplicated presentation ranged from 65.0% for patients with high cost sharing to 75.7% for patients with low cost sharing (Table 1). Additional characteristics associated with higher rates of early, uncomplicated presentation included age between 18 and 24 years, female sex, and low number of comorbidities. There was considerable geographic variation with regional rates of early, uncomplicated presentation ranging from 66.1% in the Mountain region states to 73.9% for patients residing in Middle Atlantic states. There was no clear differential rate of early, uncomplicated disease between patients living in urban compared with rural communities.

Table 2 gives multivariable logistic regression models for the primary and secondary outcomes, controlling for site of disease, patient age, patient sex, calendar year, quarter of admission, US Census Bureau geographic division, rural place of residence, and local area Social Deprivation Index. Compared with low total cost sharing, high total cost sharing was associated with significantly lower odds of early, uncomplicated presentation of acute diverticulitis or appendicitis (OR, 0.63; 95% CI, 0.60-0.65). Secondary outcomes showed that, compared with patients with low total cost sharing, patients with high cost sharing had decreased odds of receiving optimal surgical care (OR, 0.96; 95% CI, 0.93-0.99) or minimally invasive surgery (OR, 0.89; 95% CI, 0.84-0.95).

**Table 2.** Logistic Regression Models for Total Cost Sharing of Early, Uncomplicated Presentation; Optimal Surgical Care; and Minimally Invasive Surgery, 2013-2017

Total cost-sharing quartile ($)	Odds ratio (95% CI)		
	Early, uncomplicated presentation (n = 151 852)	Optimal surgical care (n = 151 852)	Minimally invasive surgery (n = 67 954)
Q1 (0-502)	1 [Reference]	1 [Reference]	1 [Reference]
Q2 (503-1725)	0.93 (0.90-0.96)	0.82 (0.79-0.84)	1.05 (0.98-1.11)
Q3 (1726-3082)	0.76 (0.73-0.78)	0.89 (0.86-0.92)	0.94 (0.88-0.99)
Q4 (>3082)	0.63 (0.60-0.65)	0.96 (0.93-0.99)	0.89 (0.84-0.95)
* P* value for trend in primary model^a^	<.001	.65	<.001

Abbreviation: Q, quartile.

^a^ Primary model (N = 151 852), adjusted for disease, age, sex, calendar year, year quarter, social deprivation index, rurality, US Census Bureau geographic division, and type of benefit plan.

In sensitivity analyses (Table 3), total cost sharing higher than $800 compared with $800 or lower indicated similar results for primary and secondary outcomes in the high (quartile 4) vs low (quartile 1) total cost-sharing quartile models. Among patients aged 25 to 64 years, higher cost sharing was associated with significantly lower odds of early, uncomplicated presentation (OR, 0.62; 95% CI, 0.60-0.65), optimal surgical care (OR, 0.96; 95% CI, 0.93-0.99), and minimally invasive surgery (OR, 0.90; 95% CI, 0.84-0.96).

**Table 3.** Sensitivity Analyses of Logistic Regression Models for Total Cost Sharing on Early, Uncomplicated Presentation; Optimal Surgical Care; and Minimally Invasive Surgery, 2013-2017

Variable	Odds Ratio (95% CI)		
	Early, uncomplicated presentation	Optimal surgical care	Minimally invasive surgery
Total cost-sharing threshold $800, $ (n = 151 852)			
0-800	1 [Reference]	1 [Reference]	1 [Reference]
>800	0.75 (0.73-0.77)	0.93 (0.90-0.95)	0.94 (0.89-0.98)
Aged 25-64 y (n = 140 571)^a^			
Total cost-sharing quartile ($)			
Q1 (0-502)	1 [Reference]	1 [Reference]	1 [Reference]
Q2 (503-1725)	0.92 (0.89-0.96)	0.81 (0.79-0.84)	1.05 (0.99-1.12)
Q3 (1726-3082)	0.75 (0.72-0.77)	0.88 (0.85-0.91)	0.94 (0.89-1.01)
Q4 (>3082)	0.62 (0.60-0.65)	0.96 (0.93-0.99)	0.90 (0.84-0.96)
Appendicitis (n = 65 424)^**a**^			
Total cost-sharing quartile ($)			
Q1 (0-502)	1 [Reference]	1 [Reference]	1 [Reference]
Q2 (503-1725)	1.05 (0.99-1.11)	1.13 (1.06-1.21)	1.03 (0.95-1.12)
Q3 (1726-3082)	0.87 (0.83-0.92)	1.44 (1.35-1.55)	0.95 (0.88-1.03)
Q4 (>3082)	0.69 (0.65-0.72)	1.44 (1.35-1.54)	0.92 (0.85-0.99)
Diverticulitis (n = 86 428)^a^			
Total cost-sharing quartile ($)			
Q1 (0-502)	1 [Reference]	1 [Reference]	1 [Reference]
Q2 (503-1725)	0.83 (0.80-0.87)	0.75 (0.72-0.78)	1.08 (0.97-1.19)
Q3 (1726-3082)	0.68 (0.64-0.71)	0.78 (0.75-0.81)	0.89 (0.80-0.99)
Q4 (>3082)	0.58 (0.55-0.61)	0.87 (0.83-0.90)	0.81 (0.73-0.90)
Elixhauser comorbidity model (n = 54 588)^b^			
Total cost-sharing quartile ($)			
Q1 (0-502)	1 [Reference]	1 [Reference]	1 [Reference]
Q2 (503-1725)	0.89 (0.84-0.94)	0.78 (0.74-0.82)	1.08 (0.97-1.20)
Q3 (1726-3082)	0.75 (0.71-0.79)	0.84 (0.80-0.89)	0.93 (0.84-1.03)
Q4 (>3082)	0.63 (0.60-0.66)	0.88 (0.84-0.93)	0.90 (0.81-0.99)

Abbreviation: Q, quartile.

^a^ Adjusted for disease, age, sex, calendar year, year quarter, Social Deprivation Index, rurality, Census Bureau geographic division, and type of benefit plan.

^b^ Study period October 2015 through December 2017; adjusted for disease, age, sex, calendar year, year quarter, Elixhauser comorbidity index, Social Deprivation Index, rurality, Census Bureau geographic division, and type of benefit plan.

Sensitivity analyses by diagnosis were consistent when evaluating high cost sharing in appendicitis or diverticulitis for early, uncomplicated presentation and for minimally invasive surgery. For the appendicitis analysis, higher cost sharing was associated with increased odds of receiving optimal surgical care (OR, 1.44; 95% CI, 1.35-1.54); for diverticulitis, higher cost sharing was associated with decreased odds of receiving optimal surgical care (OR, 0.87; 95% CI, 0.83-0.90). High cost sharing was associated with decreased receipt of minimally invasive surgery for patients undergoing surgery for either appendicitis (OR, 0.92; 95% CI, 0.85-0.99) or diverticulitis (OR, 0.81; 95% CI, 0.73-0.90). Finally, in the cohort of 54 588 patients in the study period from October 2015 to December 2017 that included adjustment for comorbidities, the results of total cost sharing for all 3 outcomes were similar to the main overall results (Table 3).

## Discussion

In this cohort study of more than 150 000 patients with acute diverticulitis or appendicitis, we found that patients with high cost-sharing plans were less likely than patients with low cost-sharing plans to present with early, uncomplicated disease. Secondary findings showed that high cost sharing was also associated with a lower likelihood of receiving optimal surgical care and minimally invasive surgery if a procedure was performed. These results were consistent even after controlling for patient comorbidities, community-level socioeconomic deprivation, and regional variation.

Recent policy changes have increased individual responsibility by using cost sharing to reduce patient care-seeking behavior. The Kaiser Family Foundation reported^27^ that, whereas benefit plans covered a significant share of employee health costs, the mean out-of-pocket spending grew 58%, an increase that outpaced the increase in employee wages during the study years from 2007 through 2017. The present study found a steady increase in high cost sharing during a 5-year study period. Subsequently, delays in seeking care for appendicitis and diverticulitis increased the likelihood of complicated presentation and, ultimately, costlier and morbid treatment. In a 2013 study by Kong et al,^28^ the authors found that the mean cost (£566, approximately US $778) among patients presenting with early, uncomplicated appendicitis was appreciably less costly to treat than those with complicated appendicitis by a factor of 2 up to a factor of 11. Costs of hospital stay, operation, anesthesia, and antibiotics were included in their cost analysis. They noted that, as the pathology progressed, the treatment costs rose exponentially and that 90% of the total cost for managing acute appendicitis was associated with advanced disease. Understanding the financial burden of acute diverticulitis was the basis for a study by Cammarota et al^29^ examining hospitalization costs for uncomplicated and complicated diverticulitis. The authors found that, for the study period from 2005 through 2015, the cost for complicated diverticulitis had increased to 3.5 times as high for patients with a surgery stay than for patients with a medical stay for uncomplicated diverticulitis. Similarly, another investigation found that patients with perforated diverticulitis are hospitalized longer (10.4 vs 8.6 days) and pay approximately $1100 more per admission than patients with uncomplicated diverticulitis.^30^ Acute appendicitis and diverticulitis are representative emergency surgery diagnoses; they are common, costly, and, most importantly, sensitive to the repercussions of care delays. These results suggest that higher cost sharing may disincentivize early presentation; thus, patients are less likely to seek care with early, uncomplicated disease and consequently are less likely to receive optimal or minimally invasive surgery. Overall, these costs of care are associated with a significant—and critically, modifiable—burden on the health care system.

There are considerable knowledge gaps in our understanding of how cost sharing influences patient presentation and subsequent management of acute surgical conditions. The data in the present study are consistent with prior medical literature, namely, the RAND Health Insurance Experiment, in which increasing cost sharing was associated with decreased health-seeking behavior for both indicated and discretionary care.^18^ Similarly, the Oregon Medicaid expansion was associated with increased use of health care services.^16^ Prior studies assessing the repercussions of the 2006 Massachusetts and 2010 health insurance expansion showed that increased insurance coverage was associated with earlier presentation of patients with acute surgical conditions and increased receipt of optimal surgical care.^20,31,32,33,34^ Our findings build on this literature to show an association between higher patient cost sharing and decreased odds of patients presenting with early, uncomplicated disease.

Our sensitivity analyses using a threshold of $800 to define the high and low cost-sharing groups showed that these results held true. This threshold was the median out-of-pocket expense for insured patients in the United States from 2016 through 2017.^35^ The value holds practical importance given that approximately 40% of the US population would struggle to pay an unexpected cost of $400.^36^ Therefore, cost differences above this threshold become more sematic than practically consequential for most of the population. Although our use of an absolute dollar amount in defining high vs low cost sharing introduces the potential for reverse causality, additional sensitivity models comparing patients with high-deductible plans were entirely consistent with our primary models. Similarly, sensitivity analyses results assessing the group of patients 25 to 64 years of age (ie, removing patients 18 to 24 years of age) were consistent with the overall sample. Since the passage of the Affordable Care Act in 2010, young adults aged 18 to 24 years are most likely to remain on parental health insurance plans until the age of 26 years, and thus health-seeking behaviors may be less sensitive to cost sharing. Our secondary outcomes showed variation in optimal surgical care, with high cost sharing associated with increased odds of receiving an appendectomy during the admission. This finding could be due to a variety of factors, including increased use of nonoperative management for less severe disease as well as for perforated appendicitis with abscesses.^37,38^ Additional work is warranted to dissect how cost sharing is associated with surgical management received for appendicitis.

### Strengths and Limitations

Several advantages and limitations may influence the depth and generalizability of our analysis in the studied population. The conditions included in this study have unique *ICD-9* and *ICD-10* codes that enabled more precise capturing of complicated presentation and optimal management.^5,6,20^ Using stringent exclusion criteria, such as erroneous coding, duplicate claims, and outlier length of stay, removed only 1% of hospitalizations. As such, we believe that these cost-sharing data are sufficient for proper statistical analysis and interpretation, and prior studies have successfully measured total cost sharing using HCCI data.^39^ Although potentially limited in terms of generalizability to patients beyond the study population and diagnoses, the HCCI data provided a unique opportunity to bridge granular claims data with clinical information in ways not possible with other administrative or registry data. We attempted to control for patient comorbidities using age and the Elixhauser comorbidity index in addition to controlling for other local area socioeconomic factors, including community-level socioeconomic deprivation. There are likely additional unobservable factors that may influence patient presentation and subsequent management. If present, however, we anticipate that this adverse selection would minimize the association between cost sharing and the measured outcomes. In addition, we recognize limitations in our secondary outcomes of optimal surgical management and minimally invasive surgery. There are numerous factors, including patient preference, surgeon experience or training, and capability of treating facility, that influence the specific care received. The association of these factors with individual patient care were not accounted for in the present analysis and may influence the care received independent of cost sharing. However, these measures capture additional downstream ramifications of our primary outcome—delayed presentation—and should be targeted for additional analyses.

We were unable to break down overall cost sharing and thus cannot speak to individual associations with deductibles, coinsurance, and copayments. Additionally, we recognize that missing or incomplete data on cost sharing may influence our study results. Furthermore, the HCCI data set lacks plan benefit data; thus, we were unable to reliably determine ex ante cost sharing. However, using the ex post measure of cost sharing provides meaningful evidence of the role of cost sharing. This is especially true in the context of the acute conditions included in this study. Each diagnosis and subsequent optimal intervention is largely nondiscretionary in terms of both patients and clinicians, and the ultimate care received is largely determined by the timing of presentation. We acknowledge that some variation in both cost and quality of care exists at the level of the hospitals treating patients. However, we were unable to adjust for the treating facility in this analysis. Although the absolute amount of cost sharing may be unknown to patients ex ante, patients are likely to have a general awareness as to whether they will expect substantial costs or if their insurance plan provides substantial coverage. Finally, it is important to recognize that our findings show only an association between cost sharing and patient presentation with complicated disease. Limitations in available data and study design prevent establishment of a causal relationship. Further prospective or quasi-experimental studies may further elucidate causality between cost sharing and patient presentation and management of surgical conditions.

## Conclusions

Ultimately, these are timely findings given growing efforts to increase cost sharing in high-deductible coverage for nongroup and individual marketplace plans.^33^ Although these policies aim to increase individual responsibility in health care behavior, it is becoming increasingly clear that the clinical and financial consequences are severe. The research conducted in this cohort showed that higher cost sharing is associated with later presentation of acute appendicitis and diverticulitis and with reduced odds of receiving minimally invasive surgery. Clinical implications may include longer hospitalizations, increased readmission risk, higher morbidity, and worse quality of life. As an increasing number of people face high out-of-pocket costs either through high-deductible employer or marketplace health plans, the risks of late presentation and potentially higher treatment costs become greater. Understanding how cost sharing influences access to prompt, high-quality surgical care for common, costly, and highly morbid medical conditions is critical to informing public and private insurance plan design. As policymakers debate the degree to which cost sharing encourages responsible health care behavior vs impeding use, empirical and up-to-date data on the real-world repercussions of the current plans may provide essential information to improve both clinical care and efficacy of coverage.

## Notes

Supplement. **eTable 1.** *ICD-9* and *ICD-10* Diagnosis Codes Used for Inclusion in Study **eTable 2.** *ICD-9* and *ICD-10* Diagnosis and Procedure Codes for Early Uncomplicated Presentation, Optimal Surgical Care and Minimally Invasive Surgery **eTable 3.** Total Cost Sharing by Patient Characteristics Among 151 852 Patients (2013-2017)
