# Supplementary material for: Association of Cost Sharing With Delayed and Complicated Presentation of Acute Appendicitis or Diverticulitis
Source: JAMA Health Forum. 2021 Sep 3;2(9):e212324. doi: 10.1001/jamahealthforum.2021.2324 (PMC8796960; doi:10.1001/jamahealthforum.2021.2324)
Supplement: Supplement. — eTable 1. ICD-9 and ICD-10 Diagnosis Codes Used for Inclusion in Study eTable 2. ICD-9 and ICD-10 Diagnosis and Procedure Codes for Early Uncomplicated Presentation, Optimal Surgical Care and Minimally Invasive Surgery eTable 3. Total Cost Sharing by Patient Characteristics Among 151 852 Patients (2013-2017) [file jamahealthforum-e212324-s001.pdf]

Supplementary Online Content

Loehrer AP, Leech MM, Weiss JE, et al. Association of cost sharing with delayed and complicated presentation of acute appendicitis or diverticulitis. *JAMA Health Forum*. 2021;2(9):e212324. doi:10.1001/jamahealthforum.2021.2324

eTable 1. *ICD-9* and *ICD-10* Diagnosis Codes Used for Inclusion in Study

eTable 2. *ICD-9* and *ICD-10* Diagnosis and Procedure Codes for Early Uncomplicated Presentation, Optimal Surgical Care and Minimally Invasive Surgery

eTable 3. Total Cost Sharing by Patient Characteristics Among 151 852 Patients (2013-2017)

This supplementary material has been provided by the authors to give readers additional information about their work.

© 2021 Loehrer AP et al. *JAMA Health Forum*.

eTable 1. *ICD-9* and *ICD-10* Diagnosis Codes Used for Inclusion in Study

Diagnosis	ICD-9 Diagnosis Codes	ICD-10 Diagnosis Codes
Acute Appendicitis	540.0, 540.1, 540.9, 541, 542	K35.2, K35.20, K35.21, K35.3, K35.30, K35.31, K35.32, K35.33, K35.8, K35.80, K35.891, K36, K37
Acute Diverticulitis	562.10, 562.11, 562.12, 562.13	K57.2, K57.20, K57.21, K57.3, K57.30, K57.31, K57.32, K57.33

Abbreviations: ICD-9 International Classification of Diseases, 9th Edition. ICD-10 International Classification of Diseases, 10th Edition

© 2021 Loehrer AP et al. *JAMA Health Forum*.

eTable 2. *ICD-9* and *ICD-10* Diagnosis and Procedure Codes for Early Uncomplicated Presentation, Optimal Surgical Care and Minimally Invasive Surgery

Early Uncomplicated Presentation	Optimal Surgical Care	Minimally Invasive Surgery*
**Absence of Diagnosis Codes**	**[1]Presence of Procedure Codes<br>[2]Absence of Procedure Codes**	**[a] Open vs [b] Laparoscopic Surgical Procedure Codes**
**[1]Acute Appendicitis**		
540.0, 540.1,<br>567.22, 567.29,<br>567.31, 567.38,<br>569.5,<br>785.50, 785.52, 995.91, 995.92<br><br>K35.2, K35.20,<br>K35.21, K35.32, K35.33,<br>K63.2, K63.20, K65.0, K65.1,<br>K68.12, K68.19,<br>R65.2, R65.20, R65.21, A41.4,<br>A41.50, A41.51, A41.52,<br>A41.53, A41.59, A41.81, A41.89<br>A41.9, R57.9, R65.20, R65.21	47.09, 47.01<br><br>0DTJ0ZZ, 0DTJ4ZZ	**a:** 47.09,<br>0DTJ0ZZ<br><br>**b:** 47.01,<br>0DTJ4ZZ
**[2]Acute Diverticulitis**		
567.31, 567.22,<br>567.29, 567.38, 567.39,<br>569.5, 569.81, 569.83,<br>785.50, 785.52, 995.91, 995.92<br><br>K57.20, K57.21,<br>K63.2, K65.1, K68.12, K68.19,<br>N32.1, N32.2, N82.3, N82.4	46.01, 46.03, 46.10, 46.11, 46.13, 46.14,<br>46.20, 46.21, 46.22, 46.23, 46.24<br><br>0DSB0ZZ, 0DSB4ZZ, 0DSB7ZZ,<br>0DSB8ZZ, 0DSBXZZ, 0D1B0Z4,<br>0D1B4Z4, 0D1B8Z4<br>0DSH0ZZ, 0DSK0ZZ, 0DSL0ZZ,<br>0DSM0ZZ, 0DSN0ZZ, 0DSP0ZZ,<br>0DSQ0ZZ, 0DSH4ZZ, 0DSK4ZZ,<br>0DSL4ZZ, 0DSM4ZZ, 0DSN4ZZ,<br>0DSP4ZZ, 0DSQ4ZZ, 0DSH7ZZ,<br>0DSK7ZZ, 0DSL7ZZ, 0DSM7ZZ,<br>0DSN7ZZ, 0DSP7ZZ, 0DSQ7ZZ,<br>0DSH8ZZ, 0DSK8ZZ, 0DSL8ZZ,<br>0DSM8ZZ, 0DSN8ZZ, 0DSP8ZZ,<br>0DSQ8ZZ, 0DSHXZZ, 0DSKXZZ,<br>0DSLXZZ, 0DSMXZZ, 0DSNXZZ,<br>0DSPXZZ, 0DSQXZZ, 0D1H0Z4,<br>0D1K0Z4, 0D1L0Z4, 0D1M0Z4, 0D1N0Z4,<br>0D1H4Z4, 0D1K4Z4, 0D1L4Z4, 0D1M4Z4,<br>0D1N4Z4, 0D1H8Z4, 0D1K8Z4, 0D1L8Z4,<br>0D1M8Z4, 0D1N8Z4	**a:** 45.75, 45.76, 45.79,<br>0DTM0ZZ, 0DTN0ZZ,<br>0DBM0ZZ, 0DBN0ZZ<br><br>**b:** 17.35, 17.36, 17.39,<br>0DTM4ZZ, 0DTN4ZZ,<br>0DBM4ZZ, 0DBN4ZZ

Abbreviations: ICD-9 International Classification of Diseases, 9th Edition. ICD-10 International Classification of Diseases, 10th Edition

*Among those with an appendectomy or colectomy

© 2021 Loehrer AP et al. *JAMA Health Forum*.

eTable 3. Total Cost Sharing by Patient Characteristics Among 151 852 Patients (2013-2017)

Characteristic[1]	Total N = 151,852	Total Cost Sharing Quartiles Q1 ($0 - $502) N = 37,957	Q2 ($502 - $1,725) N = 37,981	Q3 ($1,726 - $3,082) N = 37,954	Q4 (> $3,082) N = 37,960
	N (Col %)	N (Row %)			
**Diagnosis**					
Acute Appendicitis	65,424 (43.1)	14,240 (21.8)	14,929 (22.8)	17,040 (26.0)	19,215 (29.4)
Acute Diverticulitis	86,428 (56.9)	23,717 (27.4)	23,052 (26.7)	20,914 (24.2)	18,745 (21.7)
**Age Group (years)**					
18 – 24	11,281 (7.4)	2,757 (24.4)	2,601 (23.1)	2,850 (25.3)	3,073 (27.2)
25 – 34	18,459 (2.2)	3,778 (20.5)	4,228 (22.9)	5,019 (27.2)	5,343 (29.4)
35 – 44	27,637 (18.2)	6,054 (21.9)	6,938 (25.1)	7,227 (26.1)	7,418 (26.8)
45 – 54	43,770 (28.8)	10,873 (24.8)	11,079 (25.3)	11,059 (25.3)	10,759 (24.6)
55 - 64	50,705 (33.4)	14,495 (28.6)	13,135 (25.9)	11,799 (23.3)	11,276 (22.2)
**Gender**					
Male	79,517 (52.4)	18,643 (23.4)	19,288 (24.3)	20,330 (25.6)	21,256 (26.7)
Female	72,335 (47.6)	19,314 (26.7)	18,693 (25.8)	17,624 (24.4)	16,704 (23.1)
**Calendar Year**					
2013	35,407 (23.3)	8,905 (25.1)	9,899 (28.0)	9,185 (25.9)	7,418 (20.9)
2014	30,414 (20.0)	7,344 (24.1)	8,120 (26.7)	7,891 (25.9)	7,059 (23.2)
2015	28,542 (18.8)	6,980 (24.5)	7,126 (25.0)	7,201 (25.2)	7,235 (25.3)
2016	30,204 (19.9)	7,769 (25.7)	7,081 (23.4)	7,013 (23.2)	8,341 (27.6)
2017	27,285 (18.0)	6,959 (25.5)	5,755 (21.1)	6,664 (24.4)	7,907 (29.0)
**Calendar Quarter**					
Jan-Mar	36,585 (24.1)	6,802 (18.6)	8,752 (23.9)	10,288 (28.1)	10,743 (29.4)
Apr-Jun	39,471 (26.0)	9,336 (23.6)	10,077 (25.5)	9,978 (25.3)	10,080 (25.5)
Jul-Sep	39,056 (25.7)	10,338 (26.5)	10,026 (25.7)	9,525 (24.4)	9,167 (23.5)
Oct-Dec	36,740 (24.2)	11,481 (31.2)	9,126 (24.8)	8,163 (22.2)	7,970 (21.7)
**Elixhauser Comorbidity Index[2]**					
0	22,549 (41.3)	5,018 (22.2)	4,914 (21.8)	5,589 (24.8)	7,028 (31.2)
1	15,311 (28.1)	3,931 (25.7)	3,556 (23.2)	3,681 (24.0)	4,143 (27.1)
2	9,230 (16.9)	2,656 (28.8)	2,185 (23.7)	2,127 (23.0)	2,262 (24.5)
3+	7,498 (13.7)	2,818 (37.6)	1,635 (21.8)	1,494 (19.9)	1,551 (20.7)
**Social Deprivation Index Quartiles**					

© 2021 Loehrer AP et al. *JAMA Health Forum*.

Q1 (1 - 25)	49,536 (33.7)	12,515 (25.3)	12,914 (26.1)	12,340 (24.9)	11,767 (23.7)
Q2 (26 - 50)	38,850 (26.5)	9,504 (24.5)	9,840 (25.3)	9,726 (25.0)	9,780 (25.2)
Q3 (51 - 75)	32,226 (22.0)	7,482 (23.2)	7,944 (24.6)	8,359 (25.9)	8,441 (26.2)
Q4 (76 - 100)	26,181 (17.8)	6,350 (24.2)	6,263 (23.9)	6,542 (25.0)	7,026 (26.8)
**Rural**					
Urban	134,341 (91.5)	32,901 (24.5)	33,727 (25.1)	33,780 (25.1)	33,933 (25.3)
Rural	12,458 (8.5)	2,956 (23.7)	3,236 (26.0)	3,185 (25.6)	3,081 (24.7)
**Census Bureau Geographic Divisions**					
New England	5,489 (3.7)	1,654 (30.1)	1,473 (26.8)	1,313 (23.9)	1,049 (19.1)
Middle Atlantic	21,886 (14.9)	7,966 (36.4)	5,683 (26.0)	4,533 (20.7)	3,704 (16.9)
East North Central	20,556 (14.0)	5,217 (25.4)	5,328 (25.9)	5,041 (24.5)	4,970 (24.2)
West North Central	9,264 (6.3)	2,344 (25.3)	2,277 (24.6)	2,391 (25.8)	2,252 (24.3)
South Atlantic	34,614 (23.6)	8,047 (23.3)	8,816 (25.5)	8,421 (24.3)	9,330 (26.9)
East South Central	7,069 (4.8)	1,470 (20.8)	1,946 (27.5)	1,870 (26.4)	1,783 (25.2)
West South Central	26,302 (17.9)	4,397 (16.7)	6,242 (23.7)	7,754 (29.5)	7,909 (30.1)
Mountain	9,546 (6.5)	1,967 (20.6)	2,144 (22.5)	2,429 (25.4)	3,006 (31.5)
Pacific	12,065 (8.2)	2,788 (23.1)	3,052 (25.3)	3,214 (26.6)	3,011 (25.0)
**High Deductible Plan**					
No	113,186 (74.9)	30,782 (27.2)	30,569 (27.0)	28,157 (24.9)	23,678 (20.9)
Yes	37,959 (25.1)	6,938 (18.3)	7,302 (19.2)	9,594 (26.3)	14,125 (37.2)
**Benefit Plan Type**					
Point of Service	104,737 (69.0)	22,808 (21.8)	27,256 (26.0)	29,218 (27.9)	25,455 (24.3)
Preferred Provider Organization	17,804 (11.7)	4,724 (26.5)	4,112 (23.1)	3,627 (20.4)	5,341 (30.0)
Health Maintenance Organization	19,200 (12.6)	6,820 (35.5)	4,255 (22.2)	3,227 (16.8)	4,898 (25.5)
Exclusive Provider Organization	8,926 (5.9)	3,022 (33.9)	2,154 (24.1)	1,735 (19.4)	2,015 (22.6)
Other[3]	1,185 (0.8)	583 (49.2)	204 (17.2)	147 (12.4)	251 (21.2)

[1]Missing (N): SDI (5,059); Rurality (5,053); Census Bureau Geographic Divisions (5,061); High Deductible Plan (707)
[2]Study cohort (36.0%) for ECI (N = 54,588); Study time period: 10/2015-2017
[3]Other includes Indemnity, Short Term and Other

© 2021 Loehrer AP et al. *JAMA Health Forum*.
